# The effect of dexamethasone on post-tonsillectomy nausea, vomiting and bleeding

**DOI:** 10.1590/S1808-86942011000300017

**Published:** 2015-10-19

**Authors:** Jochen P. Windfuhr, Yue-Shih Chen, Evan J. Propst, Christian Güldner

**Affiliations:** 1 Professor; M.D. (Vice Chair); 2 M.D. (Private Practice); 3 M.D. (Resident); 4 M.D. (Resident); Department of Otorhinolaryngology, Plastic Head and Neck Surgery;Malteser Krankenhaus St. Anna; Albertus Magnus Str. 33; Duisburg, Germany

**Keywords:** dexamethasone, hemorrhage, postoperative complications, tonsillectomy

## Abstract

It has been stated, that the administration of Dexamethasone has an impact on the morbidity following tonsillectomy.

**Objective:**

To re-calculate the blood values for Dexamethasone when given as fixed doses and to evaluate the effect of Dexamethasone on post-operative nausea, vomiting and bleeding rates following tonsillectomy.

**Materials and methods:**

The charts of 272 children (2–15 years) who had undergone tonsillectomy were analyzed. The rates of post-operative nausea, vomiting and bleeding in relation to Dexamethasone were calculated-in general and different doses (0 mg/kg, <0.15 mg/kg, >0.15 mg/kg).

**Study design:**

Retrospective cohort study.

**Results:**

Dexamethasone was administered in 121 children (43.7%) according to the preference of the anesthesist (mean dose: 0.2 +/- 0.12 mg/kg; range: 0.04 - 0.62 mg/kg). There was no significant difference in nausea and vomiting (*p*=0.953) or bleeding (*p*=0.827) across groups receiving or not receiving Dexamethasone. Stratification into three different groups of Dexamethasone concentration also did not identify a dose-related risk of postoperative nausea or vomiting (*p*=0.98) or bleeding (*p*=0.71).

**Conclusion:**

At least under common non-controlled conditions in the clinic, Dexamethasone does not appear to have an effect on nausea or vomiting or bleeding following tonsillectomy.

## INTRODUCTION

Several meta-analyses investigating morbidity following tonsillectomy have demonstrated that under controlled conditions a single intravenous dose of Dexamethasone (DX) is an effective, safe and inexpensive method of reducing the incidence of postoperative nausea and vomiting (PONV) following tonsillectomy in children^1^, ^2^, ^3^, ^4^, ^5^. Unfortunately, there is substantial variability across these studies with respect to design, surgical technique, method of acquiring hemostasis and age of patients. Recently, Czarnetky et al^6^ identified a dose-dependent increased rate of post-tonsillectomy hemorrhage following administration of DX. In their study, 50 children were given either a placebo or one of three different concentrations of DX (0.05, 0.15 or 0.5 mg/kg). None of the children in the placebo group required surgical intervention for hemostasis, whereas children in all other DX groups required surgical intervention for hemostasis (5.7%; 2%; 8%, respectively).

At the primary investigator's institution, the decision to administer DX during tonsillectomy is determined by the personal anesthetist's preference. The goal of the present study was to determine the effect of DX on PONV and bleeding following tonsillectomy from a clinical situation thus maybe better mimic what effect routinely given Dexamethasone would have in ordinary praxis. We therefore calculated the exact blood values for DX related to the individual patient's weight in this study to clarify, whether or not the values vary significantly in this patient population. This would limit the value of conclusions drawn from studies with administration of DX in fixed doses. Moreover, we hypothesized that the administration of DX would result in a lower rate of PONV but not to cause an increased rate of bleeding.

## MATERIAL AND METHODS

The study was in accordance with the requirements of the associated IRB. The charts of 385 continuous pediatric patients (age < 15 years) who had undergone tonsillectomy, with or without adenoidectomy between January 2008 and December 2009 were reviewed. Children who had undergone partial (intracapsular) tonsillectomy (n=108) or who had received methylprednisolone instead of DX (n=5) were excluded from the analysis. Included patients (n=272) were otherwise healthy without a predisposition to bleeding, such as immunodeficiency, diabetes, gastritis, peptic ulcer, cardiovascular, arterial hypertension, malignant or renal diseases or on therapy with corticoids, anti-emetics, anti-histaminics or aspirin.

Patients were premedicated with oral midazolam 0.5 mg/kg 20 minutes before transportation to the operating theatre. All procedures were performed under general anesthesia with oral intubation. Anesthesia was induced with one of three volatile agents (isoflurane, sevoflurane, desflurane). An intravenous line was placed and lactated Ringer's solution infused after the eyelash reflex had disappeared. Endotracheal intubation was facilitated by 0.1 mg/kg pancuronium. All children received paracetamol 15 mg/kg per rectum. Fentanyl was given according to the preference of the anesthesist. Anesthesia was maintained with volatile agents or propofol with oxygen and N_2_O. Adrenalin-containing local anesthetic agents were never used intraoperatively. Tonsillectomies were performed using a cold technique, dissecting the tonsils with scissors, raspatory and removing the inferior pole with a snare. Hemostasis was obtained with suture ligation and bipolar cautery where required. DX was administered per each anesthetist's preference on a case-by-case basis. Patients were given either one half (4 mg) or a full (8 mg) vial of DX. The dose of DX administered was retrospectively calculated for each patient (mg/kg).

Children were extubated in the operating theatre and then transported to the postanesthesia care unit when they were awake and responded to verbal stimuli. They were closely monitored for 2 hours and transported to the ward when vital signs were stable, pain was controlled, and they did not have vomiting or bleeding. Piritramid was administered in 103 children on demand for additional pain control in the postoperative care unit.

Patients were admitted for approximately four days. Episodes of PONV and bleeding were documented.

Statistical analysis was carried out using SPSS statistical package (SPSS 16.0.2 for Windows). Non-parametric data were analyzed using Chi-square-test for categorical data and Mann-Whitney-U-test for continuous data with significance determined as *p* <0.05. In addition to investigating the effect of previously reported cut-off doses of DX on PONV and bleeding following tonsillectomy, we attempted to determine a cut-off dose of DX above which the risk of PONV or bleeding was increased.

## RESULTS

There were 272 children included in this study (129 male, 143 female). The mean age of patients was 7.43 +/-4.3 years (range 2 - 15 years). DX was administered to 121 (43.7%) children either as one half or a full vial. The mean dose of DX given (calculated retrospectively based on weight) was 0.2 +/- 0.12 mg/kg (range 0.04 - 0.62 mg/kg). Five children who were excluded from this study received methylprednisolone in high doses (100 or 250 mg) instead of DX. Nausea or vomiting was experienced by 62 of 272 (22.8%) patients, 28 of whom (45.2%) had received DX. There was no significant difference in nausea or vomiting across groups receiving or not receiving DX (*p*=0.953). Further analysis revealed that 6 of 272 patients (2.2%) had nausea without vomiting, 4 of whom had received DX (doses were 0.05, 0.12, 0.15 and 0.17 mg/kg). There was no difference in nausea across groups receiving or not receiving DX (Chi-square test, *p*=0.827). 56 of 272 patients (20.6%) had vomiting without nausea, 24 of whom (42.9%) had received DX (mean dose 0.16 +/-0.09 mg/kg, range 0.07 - 0.44 mg/kg). There was no difference in vomiting across groups receiving or not receiving DX (Chi-square test, *p*=0.953). Evaluation of the effect of DX dose (0 *vs*. <0.15 *vs*. ≥0.15 mg/kg) on postoperative nausea or vomiting did not reveal a difference across groups (Chi-square test, *p*=0.98; Table 1 and 2). 79 children received fentanyl 1μg/kg while 198 children did not. Anesthesia was maintained with volatile agents and oxygen as single agents to maintain anesthesia in 135 children with (n=220) or without (n=57) N_2_O. Maintenance of anesthesia was achieved with propofol in 142 patients, with (n=118) or without (n=24) volatile agents, again, with (n=98) or without (n=44) N_2_0. 229 of the 272 children received piritramid perioperatively, of whom 104 had received DX (45.4%). Piritramid was not administered in 48 of the 272 patients of whom 22 had received DX (45.8%). The effect of DX to prevent nausea was statistically insignificant (*p*=0.827; Chi-square-test). There was no significant association of postoperative nausea and/or vomiting and anesthetic drugs such as fentanyl (*p*=0.07), piritramid (*p*=0.026) or N_2_0 (*p*=0.406).

**Table 1:** Age distribution in three subpopulations according to specified DX values

age (years)	no DX	DX < 0,15 mg/kg	DX ≥ 0,15 mg/kg	total
1	1 (50%)	0 (0%)	1 (50%)	2
2	7 (70%)	2 (20%)	1 (10%)	10
3	23 (55%)	12 (26%)	7 (19%)	42
4	25 (63%)	12 (30%)	3 (7%)	40
5	19 (48%)	12 (30%)	9 (22%)	40
6	6 (35%)	6 (35%)	5 (30%)	17
7	6 (46%)	4 (31%)	3 (23%)	13
8	5 (36%)	2 (14%)	7 (50%)	14
9	7 (63%)	3 (27%)	1 (10%)	11
10	2 (25%)	3 (38%)	3 (37%)	8
11	4 (50%)	2 (25%)	2 (25%)	8
12	5 (42%)	6 (50%)	1 (8%)	12
13	5 (50%)	5 (50%)	0 (0%)	10
14	15 (68%)	4 (18%)	3 (14%)	22
15	21 (91%)	2 (9%)	0 (0%)	23
total	n = 151 (55%)	n=75 (28%)	n=46 (17%)	n=272

Three subgroups were compared according to the calculated concentration of dexamethasone vs. age.

**Table 2:** Incidence of PONV with specified DX values

	no DX	DX< 0,15 mg/kg	DX≥ 0,15 mg/kg
mean (mgDX/kg)	0	0.10	0.26
median (mgDX/kg)	0	0.11	0.20
std (±mgDX/kg)	0	0.03	0.13
min; max (mgDX/kg)	0	0.04; 0.14	0.15; 0.62
Ø PONV	117 (77%)	58 (77%)	35 (78%)
PONV	34 (23%)	17 (23%)	11 (22%)
total	n = 151	n=75	n=4

Comparison of the three subgroups 0 vs. <0.15 and >0.15 mg/kg could not identify a dose-related risk of or nausea and vomiting that reached statistical significance (*p*=0.98).

Hemorrhage from the tonsillectomy site occurred in 22 patients, surgical intervention was required in 17 of 272 patients (6.3%), 7 of whom (41.2%) had received DX (table 3). Primary bleeding (<24 h) was documented in 7 cases and secondary bleeding (>24 h) in 10 children. Two children had more than one episode of secondary bleeding (postoperative day 7/12 and 8/10, respectively). One of these children had received 0.15 mg DX/kg, the second no DX. There was no difference in post-tonsillectomy hemorrhage across groups receiving or not receiving DX (*p*=0.827). Evaluation of the effect of DX dose on postoperative tonsillectomy hemorrhage did not identify a dose-related risk of postoperative bleeding (*p*=0.711; table 4). We were unable to determine a cut-off dose of DX above which the risk of PONV or bleeding was increased due to the large variability of DX doses administered.

**Table 3:** Post-tonsillectomy hemorrhage vs. DX doses

Patient	age	DX (mg/kg)	PONV	PTH (day)
1.	4	0,4		day of surgery
2.	3	0,11		day of surgery
3.	15	0		day of surgery
4.	9	0	Yes	day of surgery
5.	5	0		day of surgery
6.	4	0,14		day of surgery
7.	8	0		day of surgery
8.	14	0		1.
9.	5	0,09		2.
10.	7	0,09		3.
11.	13	0,061		3.
12.	8	0		3.
13.	11	0		5.
14.	9	0		6.
15.	3	0	Yes	7. + 12.
16.	3	0,15		8. + 10.
17.	8	0		9.
18.	12	0.19		8.*
19.	14	0		1.*
20.	15	0		1.*
21.	3	0.62		7.*
22.	6	0.15		5.*

DX=dexamethasone; PONV=postoperative nausea and vomiting; PTH = post-tonsillectomy hemorrhage;

* =no surgical intervention required

**Table 4:** Incidence of postoperative bleeding with specified DX values

	no DX	DX< 0,15 mg/kg	DX≥ 0,15 mg/kg
Uneventful	139 (92%)	70 (93%)	41 (89%)
postoperative bleeding	12 (8%)	5 (7%)	5 (11%)
Total	n = 151	n=75	CDiiC

Stratification into three different groups of DX concentration (0 vs. <0.15 and >0.15 mg/kg) could not identify a dose-related risk of postoperative bleeding (*p*=0,711).

## DISCUSSION

Previous studies investigating the effect of intravenously administered DX on nausea and vomiting following tonsillectomy have found promising results^2^, ^6^, ^7^, ^8^, ^9^, ^10^, ^11^, ^12^, ^13^, ^14^, ^15^, ^16^, ^17^, ^18^, ^19^, ^20^ In these studies, DX was most commonly administered as a single intravenous dose before tonsil dissection. In these studies, administered doses were 4^21^, 8^22^,10^23^ or 20 mg^24^ or the doses were related to the body weight ranging from 0.15^25^, ^26^, ^27^, 0.5^6^, ^13^, ^14^, ^18^, ^19^, ^28^, ^29^, 0.7^30^ to 1^7^, ^9^, ^10^, ^17^, ^31^, ^32^ mg/kg. In some studies, a dose of 8^11^, ^13^, ^26^, ^29^, ^33^, 10^10^, 12^28^, ^34^, 15^31^, 16^9^, ^19^, 20^6^, 25^7^, ^30^, 24^32^ or 50 mg^17^ was injected with the additional goal of reducing post-tonsillectomy pain^6^, ^7^, ^9^, ^10^, ^12^, ^14^, ^15^, ^16^, ^17^, ^19^, ^20^, ^22^, ^24^, ^25^, ^35^, ^36^, ^37^, ^38^. Unfortunately, there is great variability across studies, making it difficult to draw any conclusions.

Czarnetzki et al were the first to report a dose-related increased risk of post-tonsillectomy hemorrhage following the administration of DX.^6^ Patients in this study received a placebo or one of three different concentrations of DX (0.05; 0.15 or 0.5 mg/kg). Due to the large variability in dose of DX administered in the present study, we decided first to determine if there was any effect of DX on PONV or bleeding, and then investigate the dose-response relation of DX using similar cut-off values similar to those used by Czarnetzki (0 mg/kg, <0.15 mg/kg, >0.15 mg/kg). In contrast to standardized but somewhat artificial conditions of contemporary studies, our case-controlled study evaluated outcomes following tonsillectomy under common clinical conditions. Because guidelines regarding the administration of DX are not yet standardized at our institution and the decision to administer DX is at the discretion of the anesthetist, a retrospective case-controlled study could be conducted. The present study included 272 children with a mean age of 7.43 years which compares to the majority of studies published in the literature (table 4). When administered, the mean dose of DX was 0.2 mg/kg with a range of 0.04 to 0.62 mg/kg per dose. Those authors^20^, ^21^, ^22^, ^23^, ^24^ who administered DX not in relation to weight but as a fixed dose did not calculate the effective dose. The results of the present study indicate that the effective dose is significantly influenced by weight even in a pediatric population which compounds comparison with more sophisticated studies. The median effective dose of 0.2 mg/kg may be lower than calculated in most of the studies (table 4). However, evidence has been given, that higher blood concentrations are not associated with an increased benefit^32^.

The rate of PONV in the present study was 23% with or without DX. This rate is lower than reported in comparable groups (table 5). There was no difference in nausea or vomiting across groups receiving or not receiving DX in the present study. This finding is supported by a number of well-designed prospective studies^25^, ^28^, ^37^, ^39^, ^40^ and refuted by others^25^, ^28^, ^37^, ^39^, ^40^. For example, results contrast with Karaman who found a dose-dependent decrease in PONV rates following administration of DX^30^. Even though DX dose levels in the present study (0.62 mg/kg) did not attain concentrations of as high as those administered by Karaman (0.7 mg/kg), doses in the present study were close enough that one would expect to see some effect. This reasoning is supported by Kim et al^31^ who verified in a double-blinded, prospective randomized study on 125 children that DX doses of 0.0625 mg/kg, 0.125, 0.25, 0.5 and 1 mg/kg are equally effective in reducing PONV rates.

**Table 5:** Post-tonsillectomy hemorrhage and vomiting in the literature (sorted by date)

Author	age (DX; control group)	N (DX; control group)	bleeding rate (DX; control group)	PONV rate (DX; control group)	DX dose	dissection technique	hemostasis technique
Catlin^44^	4-6; 5-12	10; 15	20%; 6,7%	ns	8 mg/m2	cold	E
Volk^23^	6.9; 7.0	25; 24	8.0%; 4.2%	ns	10 mg	cold	E
Tewary^45^	22; 21 (median)	40; 42	0%	ns	4 mg	cold	suture ligation
Fields^22^	24.7; 23.7	29; 29	0%	ns	8 mg	cold	suture ligation
Ohlms^28^	7.0; 6.9	34; 35	8.8%; 0%	35%, 43%	0.5 mg/kg; max. 12 mg	cold	E
Splinter^26^	6.9; 6.9	63; 70	0%	40%; 71%	0.15 mg/kg;max. 8 mg	ns	ns
April^9^	6.5; 7.2	41; 39	2.4%; 2.6%	4.8%; 25.6%	1 mg/kg; max. 16 mg	E	E
Tom^10^	1-18 (mean: 5.64)	26; 32	3.8%; 6.3%	4%; 48%	1 mg/kg; max. 10 mg	E	E
Pappas^7^	6.0; 5.8	63; 65	0%	48%; 88%	1 mg/kg; max. 25 mg	E	E
Carr^24^	26.9; 27.6	15; 14	6.7%; 14.3%	ns	20 mg	E	E
Vosdoganis^11^	5.0; 5.7	22;19	0%	45%; 63%	0.4 mg/kg; max. 8 mg	ns	ns
Aouad^13^	5.1; 4.6	53; 53	0%	23%; 51%	0.5 mg/kg; max. 8 mg	E	E
Stewart^46^	>16	104; 48	17.3%; 27.1%	23%; 30%	8 mg, 2 mg postop; 2x2 mg day 1-4; 2 mg day 5-8	E	ns
Giannoni^47^	(3-15)	25; 25	8%; 4%	28%; 36%	1.0 mg/kg; max. 16 mg	E after LA	E
Elhakim^33^	5.2; 5.1	55; 55	0%	20%; 56%	0.5 mg/kg; max. 8 mg	E	E
Al-Shehri^16^	18-35	15; 15	0%	0%; 13%	3 × 8 mg/24 h	E	E
Hanasono^17^	5.7; 5.9	106; 113	2.8%; 0.9%	1.2; 2.1 episodes of emesis	1 mg/kg; max. 50 mg	Ecold	E
Celiker^48^	5.3; 6.1; 5.9 vs. 5.8	26; 27; 24 vs. 25	0%	27%; 37%; 29% vs. 36%	0.15; 0.1; 0.05 vs. 0 mg/kg; max. 8 mg	cold	suture ligation
Samarkandi^18^	7.2; 7.2	29; 31	0%	37.9%; 74.2%	0.,5 mg/kg	E	E
Malde^25^	12; 15	45; 45	0%; 1.1%	22%; 29%	0.15 mg/kg	cold	suture ligation
McKean^20^	23; 26	24; 22	0%	29%; 77%	10 mg	cold	suture ligation
Kaan^19^	7.6; 9.3	32; 30	0%	19%; 33%	0.5 mg/kg; max. 16 mg	cold	suture ligation
Fazel^29^	9.5; 10.1	50; 50	0%	25%; 62	0.5 mg/kg; max. 8 mg	ns	ns
Czarnetzki^6^	6; 5; 6 vs. 6 (median)	53; 54; 52 vs. 54	11%; 4%; 24% vs. 4%	38%; 24%; 12% vs.44%	0.05; 0.15; 0.5 vs. 0 mg/kg; max. 20 mg	cold or E after LA	suture ligation or E
Karaman^30^	(5.9)	100; 50	0%	8%; 4%; 80%	0.2 mg/kg; 0.7 mg/kg; max. 25 mg	ns	ns
this study	7.0; 7.78	126; 151	7.9%; 7.9%	22.5%; 22.2%	2 mg (<20 kg) 4 mg(>20 kg)	cold	suture ligation

E=electrosurgery; cold=dissection of the tonsils with raspatory, scissors; LA=local infiltration of local anesthetics prior to tonsil dissection; ns=not stated; age in brackets indicates that ages of the subgroups were specified;

The post-tonsillectomy hemorrhage rate requiring surgical intervention in the present study was 6.3%. When the five patients who experienced minor episodes of bleeding were included, the post-tonsillectomy bleed rate increased to 8.1%. Rates in the literature vary significantly (table 4) which may also result from different surgical techniques, length of follow-up and definitions of post-tonsillectomy hemorrhage. The rate of post tonsillectomy hemorrhage in the present study is higher than reported previously^41^, ^42^ which appears attributable to the use of electrosurgery either for dissection or hemostasis. There was no difference in post-tonsillectomy hemorrhage rates across groups receiving or not receiving DX in the present study which is only partly supported by other authors (table 4).

Postoperative vomiting was associated in only 1 of the 22 cases with major bleeding (5.4%) and in no case with minor bleeding suggesting that vomiting itself is not likely a risk factor for post-tonsillectomy hemorrhage. This contrasts with previous reports of vomiting leading to excessive hemorrhage^43^.

The present study has some limitations which have to be considered. First, it is a retrospective observational study. Although it reports from a clinical situation and thus maybe better mimic what effect routinely given Dexamethasone would have in ordinary praxis its weakness is, that other factors that can influence PONV are not controlled. However, multivariate ANOVA-analysis was unable to detect dexamethasone or one of the various anesthetic agents as a risk factor for PONV in our study population (data not shown). Second, the large amount of variability in the dose of DX administered. In an attempt to overcome this limitation, doses of DX were stratified into three groups for analysis. Even though stratification values were selected based on previously reported values, analysis based on different cut-off values may yield different results. We were unable to determine a cut-off dose of DX above which the risk of PONV or bleeding was increased due to the large variability of DX doses administered.

## CONCLUSION

The administration of fixed doses of Dexamethasone results in significant differences of blood values (factor 15.5). At least under non-controlled conditions in the clinic, Dexamethasone appears not to have an effect on nausea or vomiting or bleeding following tonsillectomy.
